# Pilot Study on Gait Classification Using fNIRS Signals

**DOI:** 10.1155/2018/7403471

**Published:** 2018-10-17

**Authors:** Hedian Jin, Chunguang Li, Jiacheng Xu

**Affiliations:** ^1^Key Laboratory of Robotics and System of Jiangsu Province, School of Mechanical and Electric Engineering, Soochow University, Suzhou, China; ^2^Collaborative Innovation Center of Suzhou Nano Science and Technology, Suzhou, China

## Abstract

Rehabilitation training is essential for motor dysfunction patients, and the training through their subjective motion intention, comparing to passive training, is more conducive to rehabilitation. This study proposes a method to identify motion intention of different walking states under the normal environment, by using the functional near-infrared spectroscopy (fNIRS) technology. Twenty-two healthy subjects were recruited to walk with three different gaits (including small-step with low-speed, small-step with midspeed, midstep with low-speed). The wavelet packet decomposition was used to find out the main characteristic channels in different motion states, and these channels with links in frequency and space were combined to define as feature vectors. According to different permutations and combinations of all feature vectors, a library for support vector machines (libSVM) was used to achieve the best recognition model. Finally, the accuracy rate of these three walking states was 78.79%. This study implemented the classification of different states' motion intention by using the fNIRS technology. It laid a foundation to apply the classified motion intention of different states timely, to help severe motor dysfunction patients control a walking-assistive device for rehabilitation training, so as to help them restore independent walking abilities and reduce the economic burdens on society.

## 1. Introduction

Population aging is a prominent problem in today's society. In 2016, approximately 12% of the world's population was over the age of 60, and this percentage would rise to approximately 21% of the world's population by 2050 [1]. Aging leads to a significant decline in elderly body movement [2] and increase of body vulnerability. These would result in the probability of fractures or other accidents increased, causing severe motor dysfunction [3–5]. Similarly, spinal cord injury (SCI) is a common disease frequently resulting in severe motor dysfunction, forcing patients to depend on a wheelchair for mobility [6]. Moreover, the number of severe motor dysfunction victims of traffic accidents and accidental injuries has also increased rapidly as society develops. As a result, above patients often remain bedridden for extended periods of time, causing some complications and increasing the probability of degeneration of bodily functions. These problems would cause serious impact on rehabilitation and impose serious economic burdens on society [7]. And the recovery of walking function is a primary desire of these patients [8]. Therefore, it is greatly meaningful to provide these patients with appropriate training to help them restore their walking ability.

However, most training instruments are passive-controlled. This leads to insufficient participation of patients and unobvious rehabilitation effect. Husemann et al. [9] conducted a controlled experiment on robotic training so as to motivate the initiative of subjects and conventional physiotherapy and found that walking ability was significantly improved by the robotic training. Veneman et al. [10] and Riener et al. [11] developed different strategies of orthotic devices to elicit greater voluntary participation of the subjects in the rehabilitation process, and it performed more effectively than a fixed repetitive pattern. Moreover, other studies had also demonstrated that training based on patient-active awareness can be more effective than passive for rehabilitation [12–14]. Therefore, the multitraining modes and patients' active participation play an important role for advancing rehabilitation. And the biomechanical information and brain information can be used to judge subjects' motion intention so as to control a walking-assistive equipment to do rehabilitation training.

Progress has been made in recent years in using identified motion intention to control walking-assistive equipment, based on biomechanical information [15–18]. Lee and Sankai [15] and Hayashi et al. [16] used the lower limbs' electromyography (EMG) signal to identify the subjects' motion intention, to control walking-assistive equipment to drive movement. Limb-movement information and foot-pressure data that were tested by foot-pressure and angle-acceleration sensors were used to identify the motion intention of subjects, to control walking-assistive equipment [16–18]. These related studies can help patients control the exoskeleton to help them carry out rehabilitation training through their motion intention and have a better recognition accuracy. However, for these severe motor dysfunction patients, biomechanical signals were very weak or abnormal, and it was also very difficult to collect. Therefore, brain information can be used to identify the patients' motion intention to help them control the walking-assistance equipment to complete independent rehabilitation training.

In recent years, several studies have investigated brain activity based on electroencephalography (EEG) signals during walking [19–26]. According to an EEG signals' mu and *ß* rhythms, three different walking speed levels were identified, with an average classification accuracy of 72.7% [22], this study also provides insight on the cortical involvement in human gait control and represents a step towards a brain-machine interface for poststroke gait rehabilitation [22]. Zhang et al. [23] used the multiple kernel learning algorithm to simultaneously learn the relative importance of different brain areas, so as to identify the region of importance, and it demonstrated that the frontal and frontocentral regions are the most important regions in controlling the exoskeleton. In addition, some studies noted that the intensity of neural activity in the motor cortex is positively correlated with walking speed, and it also proved that different motor states have different neural activities [21–25]. However, most experiments were based on treadmill, which differ from a normal gait. Moreover, the subjects often required external stimulus before experimentation, which was also not conducive to do rehabilitation training in normal environments [19–22,24–26].

On the contrary, fNIRS technology can support continuous testing under the normal environment and without external stimuli. Kim et al. [27] and Mihara et al. [28] found that the main activation areas during the change of walking speed are sensorimotor cortex (SMC), premotor cortex (PMC), and supplementary motor area (SMA). Caliandro et al. [29] demonstrated that the concentration of blood oxygen activity in the prefrontal cortex has a positive correlation with the step length, which establishes an important basis for the identification of step length. Holtzer et al. [30] also found that the activation of the PFC area is largely associated with increasing step length. A study of the premovement consciousness of the normal start and preparation determined that the proportion of oxyHb of the PFC area and premotor cortex significantly increase [31]. These studies focus on which brain region was activated when the walking speed or step length changed, instead of status recognition, which lay a theoretical basis for this study in testing areas. In addition, fNIRS technology for the identification of similar patterns also has great application prospects. Sui et al. [32] identified three levels of bicycling speed, based on the difference of oxyHb and deoxyHb, with a corresponding classification accuracy of 74%. Hong et al. [33] identified mental arithmetic (MA), right-hand motor imagery (RI), and left-hand motor imagery (LI) with an average classification accuracy of 75.6% across ten subjects. The joint mutual information (JMI) criterion was used to extract the optimal features of hemodynamic responses, to identify three images of hand clenching associated with force and speed with a final accuracy of 76.7% [34]. Yin et al. [35] applied empirical mode decomposition to reduce the physiological noise during the task, and the intrinsic mode functions were used to extract the feature vectors, to identify the motor imagery tasks of right-hand clench force and speed, with a corresponding classification accuracy of 78.33%. Most of the fNIRS studies mainly focus on classifying the different states about upper limbs. For lower limbs, the study of different motion states of spontaneous walking is sparse, and the simultaneous classification of two-dimensional variables of walking speed and step length is still in the bank.

In this study, a method based on fNIRS signals is proposed, to identify the motion intention of two-dimensional variables of walking speed and step length simultaneously under the normal environment. During the whole experiment, in order to elicit greater voluntary participation of the subjects, all movements (the start and end of every task) were spontaneously controlled by themselves, and without external stimuli. It hopes to classify the two-dimensional states of walking speed and step length timely, based on the motion intention of movement. This study expects to apply a method to classify the motion intention of different states, so as to help patients control a walking-assistive device for rehabilitation training and let them restore independent walking abilities in the future.

## 2. Experiment Design

### 2.1. Subjects

Twenty-two healthy subjects (22 mean ± 4 years old, seventeen males and five females) of Soochow University participated in this experiment. All participants were right-handed, without neurological abnormalities and other related conditions.

### 2.2. Instrument

A FOIRE-3000 optical topography system (Shimadzu Corporation, Kyoto, Japan) [32] with eight emitters and eight detectors was used to measure the light sources of wavelengths of 830 nm, 805 nm, and 780 nm, that represent the oxygenated hemoglobin (oxyHb), total hemoglobin (totalHb), and deoxygenated hemoglobin (deoxyHb), respectively. The sampling period of hemoglobin signals was 130 ms.

### 2.3. Cortical Regions

**Figure 1:**
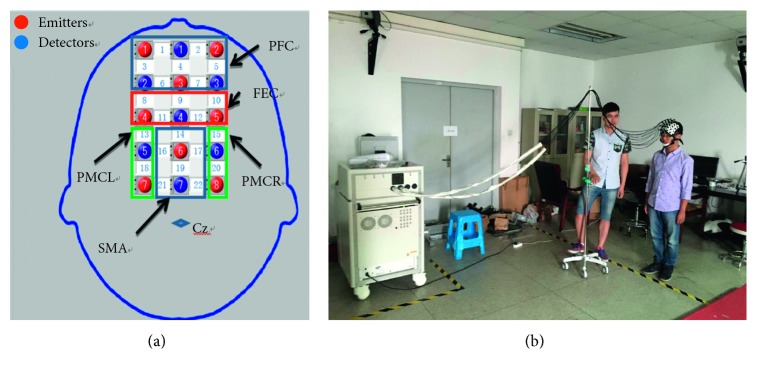
(a) The arrangement of the optodes. (b) The experimental setup. The person in the right was the subject; he was doing a rest for walking. The person in the left was a researcher who walked with the subject carrying the weight of the fNIRS cables.

Prior fNIRS research determined that the PMC, SMA, and PFC areas are largely associated with walking speed or step length [27,29–31]. In addition, the PFC area plays an important role in identifying premovement consciousness [28]. According to the international 10–20 system [36, 37] and the Brodmann partition map [38], a 3 × 5 parietal flash holder was built, which was applied to fix emitters and detectors.  Figure 1(a) shows the arrangement of the optodes, where the Cz point is the intersection of the left to right earlobe and nation to occipital tuberosity, with a distance to detector 7 of 3 cm. In the layout of the channel, the emitters 3 and 6, and detectors 1, 4, and 7 are on the connection of Nz-Cz-Lz, and the probe layout on the left and right is parallel to the connection of Nz-Cz-Lz, the distance between each detector and emitter is fixed at 3 cm. Based on the location of important region defined by above study and probe layout, it is defined that the channels 1 to 7 are in the PFC area, channels 8 to 12 are in the frontal eye cortex (FEC) area, channels 13 and 18 are in the PMC left (PMCL) area, channels 15 and 20 are in the PMC right (PMCR) area, and channels 14, 16, 17, 19, 21, 22 are in the SMA area.

### 2.4. Paradigm

The patient with very weak or no athletic ability finds it hard to undertake rehabilitation training, not to active training. According to the feedback from a rehabilitation doctor, these three walking states (contained the gait of small-step with low-speed (SL), small-step with midspeed (SM), and midstep with low-speed (ML)) were very helpful and necessary for these patients to improve their body function to restore their walking ability. Therefore, in this experiment, 22 health subjects' fNIRS signals under these three gait parameters were collected for initial research. During this experiment, because of the limited transmission lines, the walking distance was fixed at 4.4 m, and all the subjects could not exceed this range. So, the small-step was defined as approximately nine steps in fixed distance, and midstep was approximately about six or seven steps. But the walking speed depended on subjects' normal speed, and low-speed was defined that it must be slower than the normal gait obviously, about 30% to 50% of normal speed.

Before the experiment, all the subjects were asked to wash their hair, to make sure the scalp was clean. The experimental procedure and fNIRS' operating principle were also informed. And they were also told that they should maintain their heads in a steady position and their arms in a natural state when walking, without counting during the entire experiment.

**Figure 2:**
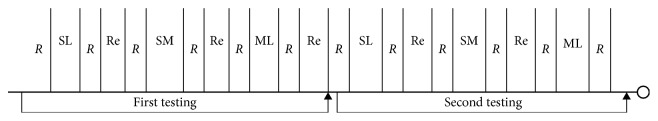
The process of the experiment. *R* represents the rest time. Re represents the backward process. SL represents the gait of small-step with low-speed. SM represents the gait of small-step with midspeed. ML represents the gait of midstep with low-speed. The first testing was a familiar process for the subjects, and the second testing was the analysis data.

Moreover, a researcher would walk with subjects carrying the fNIRS cables to reduce the effect of the cables' weight.  Figure 1(b) shows the experiment setup. All the subjects were required to train these three gait parameters, and the researcher will calculate the walking speed and step number during the walking, to ensure the gait parameter subject walked was right. However, the subject would be arranged to train these three gait parameters randomly, based on the arrangement and combination of these three states, in order to let the subject take a consideration of which gait parameter should be done during the experiment. When the subject can walk accurately, they would be told the specific process of the whole experiment. Each gait was consisted of four stages: rest, walk, rest, and retreat. During the whole experiment, each gait needed walking twice. In detail, at the beginning of the experiment, the starting point and ending point were marked previously, and all subjects stood at the starting point while resting for more than 30 s. Then, they began movement towards the ending point with the right foot. Next, the subjects did not retreat to the starting point until they stood at the ending point in a resting state for more than 30 s. The entire process of the experiment is shown in Figure 2. Moreover, the rest and start time were spontaneously controlled by the subjects. It is also stipulated that the subjects can not make a mistake in the order and gait parameters of the experiment, a researcher specializes in checking these; otherwise, the experiment will be cancelled and be redone next time. Finally, based on the feedback information after completing the experiment, the subject will take a consideration of which gait parameter they should do before walking.

## 3. Data Analysis

The totalHb and difference between the oxyHb and deoxyHb were used to extract feature vectors in the frequency domain. Eleven subjects were selected to calculate the highest recognition accuracy and its corresponding combination of feature vectors (ten for training and one for testing). When the best feature vectors combination was selected, these eleven subjects of training set were defined as training data, to calculate the final recognition accuracy of another eleven subjects. Due to various factors and maladjustments, only the second testing was used for analysis. All calculations and analysis were completed using Matlab R2016a.

### 3.1. Power Spectrum Analysis

For the eleven primary subjects in this study, power spectrum density analysis based on a rectangular window was used to analyze the rest time before and during the task segment. This method calculates the continuous frequency map of each channel in all states to determine the final analysis band and the band interval.

### 3.2. Data Preprocessing

Because the related researches on fNIRS are mainly focused on low-frequency components, over time, it will cause a zero drift in cerebral hemoglobin, significantly impacting the low-frequency component. In this study, the mathematical morphology method was proposed to remove this phenomenon [39], in order to reduce the influence of zero drift during the subsequent analysis. To identify the motion intention of all movements, 180 points before the task were analyzed. Corrosion and expansion are the main operations of this method, and their expressions are as follows, respectively:

(1) $$\begin{array}{c} \left(f\Theta k\right)\left(n\right)=\underset{m=0,...,M-1}{\min}\left\{f\left(n+m\right)-k\left(m\right)\right\};\;\;n=\left(0,\;\;1,\;\;\ldots ,\;\;N-M\right), \end{array}$$

(2) $$\begin{array}{c} \left(f⊕k\right)\left(n\right)=\underset{m=0,...,M-1}{\max}\left\{f\left(n-m\right)-k\left(m\right)\right\};\;\;n=\left(M-1,\;\;M,\;\;N-1\right), \end{array}$$

where *f*(*n*) represents the original data, (*N* − 1) is the length of the data. *k*(*m*) is a flat structure, and the length of (*M* − 1) is the number of points in ten sampling periods. Then, the opening and closing operations were calculated based on the corrosion and expansion, and their expressions are as follows, respectively:

(3) fk∘n=fΘk⊕kn,f∘kn=f⊕kΘkn.

Then, the values of performing opening operation first and then the closing operation and performing closing operation first and then the opening operation were calculated, respectively. The final result was obtained by averaging the above two values because there is a big difference between individuals, such as hair, skull thickness, etc. These lead to a difference in the signal-to-noise ratio of the collected data; therefore, the data must be normalized before the extraction as

(4) $$\begin{array}{c} xN=2*\left[\frac{x-\min}{\max-\min}\right]-1, \end{array}$$

where *x* represents an original data point in one channel and min and max represent the minimum and maximum values of all channels of the analyzed data. The *xN* represents the normalized data.

### 3.3. Feature Extraction

The results of the power spectrum analysis can confirm the decomposition layers. Then, the preprocessing data are calculated in the frequency domain by wavelet packet decomposition [40, 41]. In this study, the wavelet basis is sym4. To obtain the more obvious features, the concentration changes of all channels of the totalHb and the difference between oxyHb and deoxyHb were calculated following wavelet packet decomposition. In the time domain, the motion intention occurred at the beginning of movement. Therefore, eight points (approximately 1 s) were used for analysis. For each state, the analyzed data were stored in a matrix (M1), where the columns represented the 22 channels and the rows represented the frequency bands.

**Figure 3:**
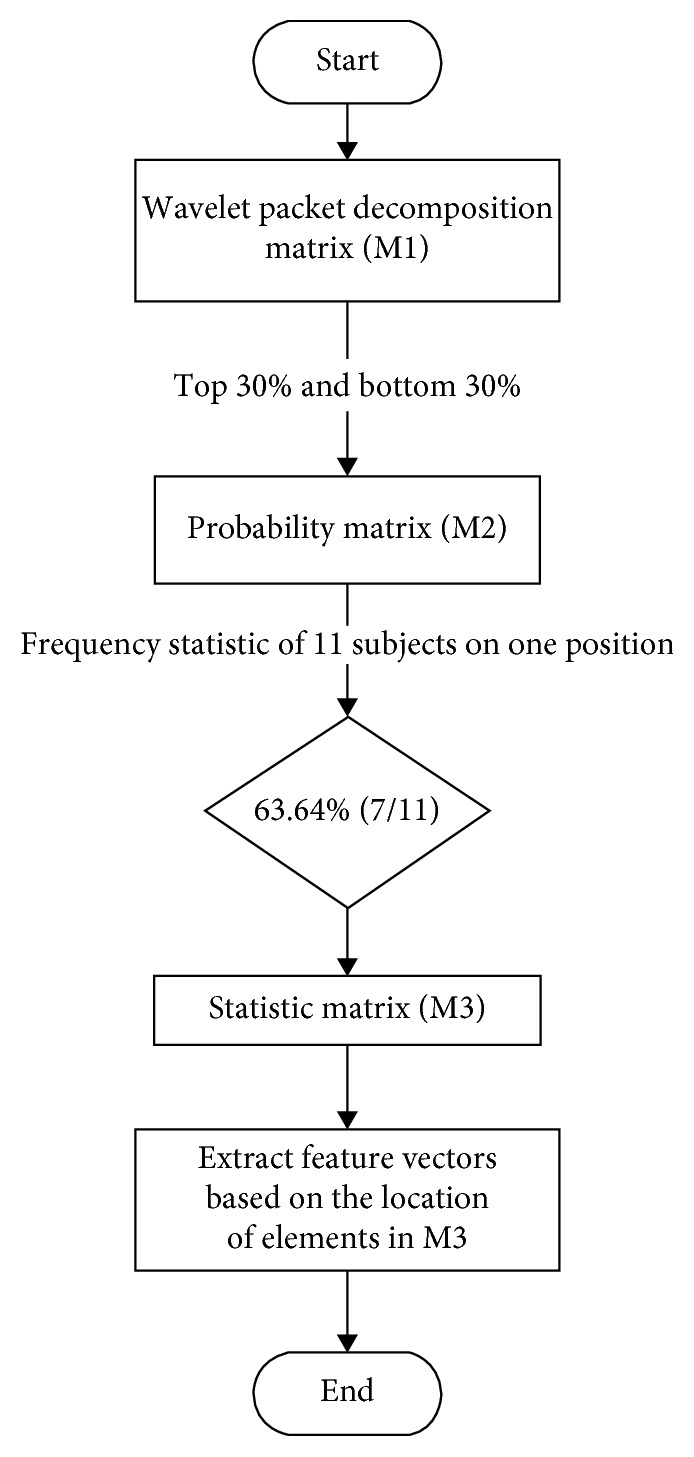
The flow of feature extraction.

For each state, each subject would admit a matrix (M1) after wavelet packet decomposition, but there is a huge difference between the values of different channels; in order to determine the significant channels and corresponding frequency bands of one state, each matrix was divided into three proportional parts based on the value of each element. In this research, the probability of 20%, 25%, 30%, 35%, and 40% were used, and based on the final accuracy of training and testing data, the probability of 30% was the best, so it was defined as the final percent. It means that the top 30% proportional parts were defined as digital “1”; the mid 40% were digital “0”; and the bottom 30% were digital “−1” (M2). For the training set, eleven subjects were selected to find out the feature vectors. In detail, based on the frequency statistic, under each state, if the frequency of same digital number on one position of these eleven matrices was seven or more (≥63.64%), this position was defined as the digital; otherwise, it was defined as digital “0” (M3). After that, the eleven matrices were combined into a new matrix under this state, which represents the features of this state. Next, the significant channels with links in frequency and space were combined to be defined as feature vectors, if the digital value was the same in this matrix. For the other states, the method of extracting of feature vectors was the same. The flow is shown in Figure 3.

### 3.4. State Classification

The libSVM algorithm [42] was used to classify these three states. To obtain the highest accuracy, the feature vectors of the totalHb and the difference between the oxyHb and deoxyHb were combined. For the primarily eleven participants, ten were selected for training data and one for testing data, a total of 11 combinations according to the different permutations. However, some feature vectors could improve the accuracy, and some could not, so all feature vectors needed requiring permutation and combination to find the best combination of feature vectors. For each of the feature vector permutations and combinations, the recognition accuracy of the 11 combinations was calculated. The final accuracy of this feature vectors combination was the average of these results. All feature vectors combinations were compared to select the highest recognition and its corresponding feature vectors. Then the eleven subjects were identified as the training data, and another eleven subjects were identified using the above feature vectors combination.

## 4. Results

### 4.1. Power Spectrum Analysis

**Figure 4:**
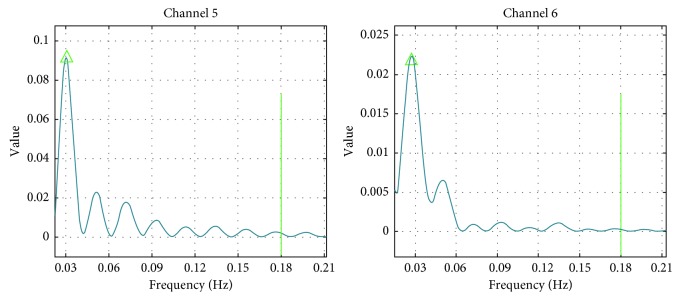
The continuous frequency maps of subject one's channel 5 and channel 6 under the ML gait of totalHb. The triangle represents the most active frequency point.

The power spectrum density analysis method could get a continuous frequency map of each channel (Figure 4). By observing 11 subjects' continuous power spectrum of each channel of the totalHb and the difference between the oxyHb and deoxyHb. It was found that the main frequency band was approximately 0 to 0.18 Hz, so it was defined as the main frequency band in this study. Moreover, it was found that the distance between two peaks was about 0.03 Hz. Therefore, 0.03 Hz is the most reasonable frequency interval, which is also associated with the number of layers of wavelet packet decomposition.

### 4.2. Data Preprocessing

**Figure 5:**
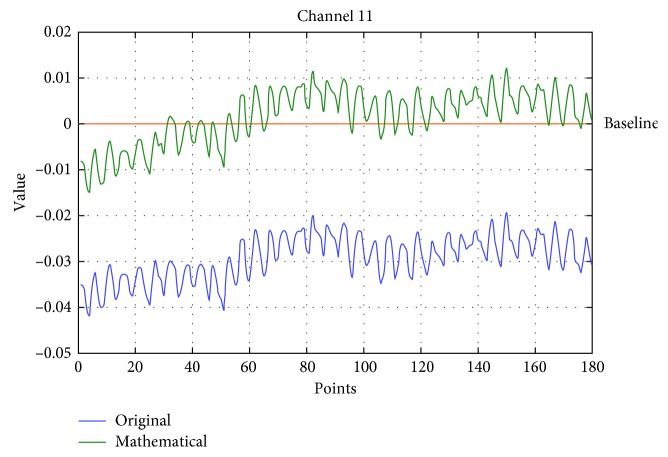
The sequence diagrams of subject one's channel 11 under the SM gait of totalHb. The blue line represents the original signal, and the green line represents the data after mathematical morphology.

The zero drift of the original data was removed using a series of operations based on mathematical morphology (Figure 5). And to highlight the key channels, the 22 channels were normalized by Formula (1). The range of all values is −1 to 1 after normalization.

### 4.3. Feature Extraction

According to the power spectrum density analysis, the main frequency band was 0 to 0.18 Hz and the frequency interval was 0.03 Hz. Because the sampling period of hemoglobin signals was 0.13 s, the signal's sampling frequency was approximately 7.7 Hz. Based on wavelet packet decomposition, the frequency band was divided into 128 groups, with each interval approximately 0.03 Hz. The first six groups (approximately 0 to 0.18 Hz) were used to extract feature vectors. The eight points at the end of the data were combined after calculating the concentration changes of all channels in the totalHb and the difference between the oxyHb and deoxyHb. For totalHb and the difference between oxyHb and deoxyHb of each subject, three 6 × 22 matrices representing the motion intention of the three gaits were created, respectively.

**Figure 6:**
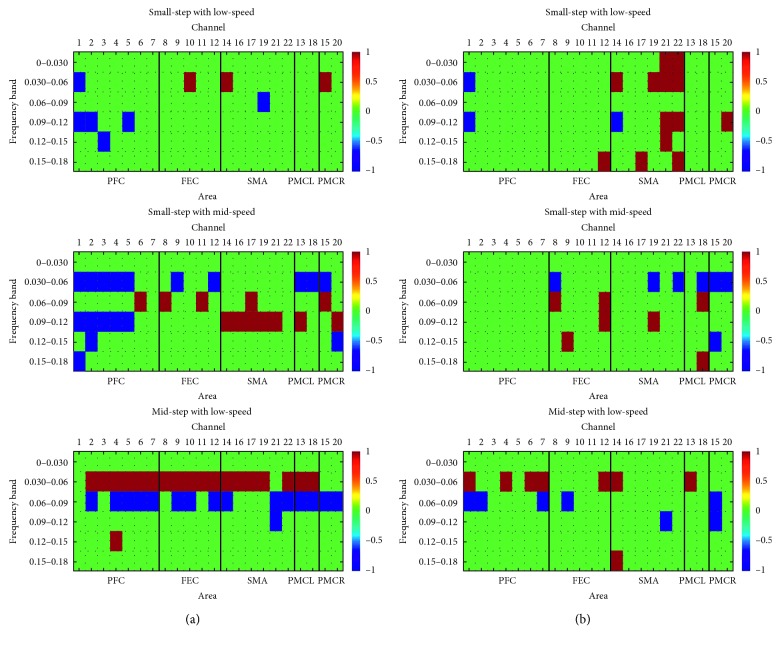
(a) The significant channels of three walking states under the total data. The red square represents digital 1: it means that the value of original seven matrices under this position has five or more under the top 30% proportion. The blue square represents digital −1: it means that the value of original seven matrices under this position has five or more under the bottom 30% proportion. The green square represents digital 0; it represents the all cases expert for above two. (b) The significant channels of three walking states under the difference between oxyHb and deoxyHb.

Based on the matrices of the last step and the above-mentioned method, the results of each state of the totalHb and the difference between oxyHb and deoxyHb are shown in Figure 6(a) and Figure 6(b). According to the spatial layout of the various channels in Figure 1(a), the channels with links in frequency or space were selected as feature vectors, if the digital value was the same. The isolated channel was not considered.

### 4.4. State Classification

For the different permutation and combination, the best average classification accuracy rate of training set was 78.79%. And under this features vector combination, the eleven training subjects were defined as the training data to classify another eleven subjects (11 subjects' second tasks × 3 states), with classification accuracy rates of 78.9% (26/33) (11 subjects' second tasks × 3 states). The recognition rates of SL, SM, and ML states are 72.72% (8/11), 72.72% (8/11), and 90.9% (10/11), respectively.

## 5. Discussion

To date, most research on lower limbs focused on which brain region is activated when the walking speed or step length changed [21–25,27,29–31]. There is little research on the identifying motion intention of lower limbs, let alone several gaits with little difference. The test environment was one of the important reasons. The fNIRS technology overcomes this restriction and can be used in the natural environment. In this study, a method based on the fNIRS signal was proposed to identify the motion intention of three similar motion states.

This study focuses on classifying the motion intention before movement of healthy subjects, and the final accuracy was 72.72% (8/11), 72.72% (8/11), and 90.9% (10/11), respectively. These results improved that the subjects' motion intention can be used to characterize the gait parameters. Moreover, through Figure 6, it can be found that there are obvious differences in the channel and frequency band under different motion intention of gait parameters. The motion intention prior to the movement initiation for patients is stronger than that for healthy subjects [43], and due to the defect of moving ability, the patient needs high attention for a certain action, whereas healthy subject does not. If one movement has been repeated for many times, it would be hard to extract the motion intention, and it will be the main influence on the classification accuracy. Therefore, the paradigm of this experiment was designed specially (Figure 2); it was that the subject can not repeat one action continuously and each movement be done only twice, and the purpose of this design is to let the subject not take a movement unthoughtfully and have a consideration before the movement. This study was still in its infancy, and through the results of healthy subjects, it lays a foundation for later research on patients' motion intention.

For patients with weak or no athletic ability, the practice of small compensation and slow pace has great practical application value [6]. In rehabilitation, the patient's active participation and coordination are important, as good rehabilitation training methods ensure that patients receive the maximum rehabilitation in the shortest possible time. This provides them with the best chance of improving their quality of life, by reducing the burden on family and society [10]. This study uses spontaneous motion intention to classify minor gait, although it is in the initial stage of study and the subjects are healthy men, but the results proved the feasibility of classifying the gait parameters through the motion intention before movement, which lays a good foundation for the patients to carry out the rehabilitation training through their motion intention and improve their walking ability in the future.

This study focused on the classification of walking intention before movement. So, all the movements were purely spontaneous, and the feature vectors used for classification were extracted before the actual movement. This method could compensate for the delay in the algorithm to communicate with external devices and lays a foundation for real-time BCI system. Although the classification accuracy was not very high, it confirmed the feasibility of controlling an exoskeleton to perform rehabilitation training for the further research.

However, there are many shortcomings in this study that need to be addressed in the future. First, the number of experiments was small, and all subjects were normal, healthy, young people. For patients with weak or no athletic ability but intact brain function, their brain function is also different from normal function, and the same is true for the elderly [23,31,44–46]. Further research is required on a large number of patients and the elderly. Second, this study focused on identifying the motion intention of different gaits. However, the method of classifying the rest time and starting awareness and the rest and ending times is a difficult task. Only these three conditions were completed. The dynamic identification was performed for real-time data to realize the real BCI system. Third, the ending point was fixed due to the limitations of the institution. The stop-awareness part was controlled by external factors. Some companies have infrared wireless devices, which may be used to realize the true spontaneous gait in the future.

## 6. Conclusions

This study presented a method of classifying the motion intention of different spontaneous gaits based on fNIRS technology. And, three different walking states were presented with final recognition rates of 78.79%. In this study, only the subjects' motion intention was used to extract the feature vectors. And this study can classify the two-dimensional gait at the same time, instead of single changes of walking speed or step length. Moreover, a combination method of permutation and combination method and libSVM algorithm is considered, all combinations of feature vectors, to reduce the influence of extraneous feature vectors on the recognition result. These results confirmed it is feasible to classify the motion intention of advanced walking by using fNIRS technology, which adds the possibility of realizing the autonomous control of walking-assistive equipment based on the BCI system.

## Data Availability

The data used to support the findings of this study are available from the corresponding author upon request.

## References

[1] Wasay M., Grisold W., Carroll W., Shakir R. (2016). World brain day 2016: celebrating brain health in an ageing population. *Lancet Neurology*.

[2] Tang W. K., Wong E., Chiu H. F. K., Lum C. M., Ungvari G. S. (2005). The Geriatric Depression Scale should be shortened: results of Rasch analysis. *International Journal of Geriatric Psychiatry*.

[3] Seichi A., Hoshino Y., Doi T. (2012). Development of a screening tool for risk of locomotive syndrome in the elderly: the 25-question geriatric locomotive function scale. *Journal of Orthopaedic Science*.

[4] Nakamura K. (2008). A “super-aged” society and the “locomotive syndrome”. *Journal of Orthopaedic Science*.

[5] Chang C. I., Chan D. C., Kuo K. N. (2011). Prevalence and correlates of geriatric frailty in a northern Taiwan community. *Journal of the Formosan Medical Association*.

[6] Rockwood K., Song X., Macknight C. (2005). A global clinical measure of fitness and frailty in elderly people. *Canadian Medical Association Journal*.

[7] Diong J., Herbert R. D., Kwah L. K., Clarke J. L., Harvey L. A. (2012). Mechanisms of increased passive compliance of hamstring muscle-tendon units after spinal cord injury. *Clinical Biomechanics*.

[8] Lapointe R., Lajoie Y., Serresse O., Barbeau H. (2001). Functional community ambulation requirements in incomplete spinal cord injured subjects. *Spinal Cord*.

[9] Husemann B., Müller F., Krewer C., Heller S., Koenig E. (2007). Effects of locomotion training with assistance of a robot-driven gait orthosis in hemiparetic patients after stroke: a randomized controlled pilot study. *Stroke*.

[10] Veneman J. F., Ekkelenkamp R., Kooij H. V. D., Kooij H. V. D., Kooij H. V. D. (2006). A series elastic- and bowden-cable-based actuation system for use as torque actuator in exoskeleton-type robots. *International Journal of Robotics Research*.

[11] Riener R., Lunenburger L., Jezernik S. (2005). Patient-cooperative strategies for robot-aided treadmill training: first experimental results. *IEEE Transactions on Neural Systems and Rehabilitation Engineering*.

[12] Jackson P. L., Lafleur M. F., Malouin F., Richards C., Doyon J. (2001). Potential role of mental practice using motor imagery in neurologic rehabilitation. *Archives of Physical Medicine and Rehabilitation*.

[13] Wolf S. L., Winstein C. J., Miller J. P. (2006). Effect of constraint-induced movement therapy on upper extremity function 3 to 9 months after stroke: the EXCITE randomized clinical trial. *Journal of the American Medical Association*.

[14] Burridge J. H., Ladouceur M. (2001). Clinical and therapeutic applications of neuromuscular stimulation: a review of current use and speculation into future developments. *Neuromodulation Journal of the International Neuromodulation Society*.

[15] Lee S., Sankai Y. Power assist control for walking aid with HAL-3 based on EMG and impedance adjustment around knee joint.

[16] Hayashi T., Kawamoto H., Sankai Y. Control method of robot suit HAL working as operator’s muscle using biological and dynamical information.

[17] Kagawa T., Uno Y. A human interface for stride control on a wearable robot.

[18] Tanabe S., Hirano S., Saitoh E. (2013). Wearable Power-Assist Locomotor (WPAL) for supporting upright walking in persons with paraplegia. *Neurorehabilitation*.

[19] Lau T. M., Gwin J. T., Ferris D. P. (2014). Walking reduces sensorimotor network connectivity compared to standing. *Journal of NeuroEngineering and Rehabilitation*.

[20] Sanctis P. D., Butler J. S., Malcolm B. R., Foxe J. J. (2014). Recalibration of inhibitory control systems during walking-related dual-task interference: a Mobile Brain-Body Imaging (MOBI) Study. *Neuroimage*.

[21] Wagner J., Solis-Escalante T., Scherer R., Neuper C., Müller-Putz G. (2014). It’s how you get there: walking down a virtual alley activates premotor and parietal areas. *Frontiers in Human Neuroscience*.

[22] Lisi G., Morimoto J. (2015). EEG single-trial detection of gait speed changes during treadmill walk. *Plos One*.

[23] Zhang Y., Prasad S., Kilicarslan A., Contreras-Vidal J. L. (2017). Multiple kernel based region importance learning for neural classification of gait states from EEG signals. *Frontiers in Neuroscience*.

[24] Presacco A., Goodman R., Forrester L., Contreras-vidal J. L. (2011). Neural decoding of treadmill walking from noninvasive electroencephalographic signals. *Journal of Neurophysiology*.

[25] Severens M., Nienhuis B., Desain P., Duysens J. Feasibility of measuring event related desynchronization with electroencephalography during walking.

[26] Gwin J. T., Gramann K., Makeig S., Ferris D. P. (2011). Electrocortical activity is coupled to gait cycle phase during treadmill walking. *Neuroimage*.

[27] Kim H. Y., Yang S. P., Park G. L., Kim E. J., You J. S. (2016). Best facilitated cortical activation during different stepping, treadmill, and robot-assisted walking training paradigms and speeds: a functional near-infrared spectroscopy neuroimaging study. *Neurorehabilitation*.

[28] Mihara M., Yagura H., Hatakenaka M., Hattori N., Miyai I. (2010). Clinical application of functional near-infrared spectroscopy in rehabilitation medicine. *Brain and Nerve*.

[29] Caliandro P., Serrao M., Padua L. (2015). Prefrontal cortex as a compensatory network in ataxic gait: a correlation study between cortical activity and gait parameters. *Restorative Neurology and Neuroscience*.

[30] Holtzer R., Mahoney J. R., Izzetoglu M., Wang C., England S., Verghese J. (2015). Online fronto-cortical control of simple and attention-demanding locomotion in humans. *Neuroimage*.

[31] Suzuki M., Miyai I., Ono T., Kubota K. (2008). Activities in the frontal cortex and gait performance are modulated by preparation. an fNIRS study. *Neuroimage*.

[32] Sui Y., Li C., Li J. Classification of desired motion speed-based on cerebral hemoglobin information.

[33] Hong K. S., Naseer N., Kim Y. H. (2015). Classification of prefrontal and motor cortex signals for three-class fNIRS-BCI. *Neuroscience Letters*.

[34] Yin X., Xu B., Jiang C. (2015). Classification of hemodynamic responses associated with force and speed imagery for a brain-computer interface. *Journal of Medical Systems*.

[35] Yin X., Xu B., Jiang C., Fu Y. (2015). NIRS-based classification of clench force and speed motor imagery with the use of empirical mode decomposition for BCI. *Medical Engineering and Physics*.

[36] Okamoto M., Dan H., Sakamoto K. (2004). Three-dimensional probabilistic anatomical cranio-cerebral correlation via the international 10-20 system oriented for transcranial functional brain mapping. *Neuroimage*.

[37] Tsuzuki D., Jurcak V., Singh A. K. (2007). Virtual spatial registration of stand-alone fNIRS data to MNI space. *Neuroimage*.

[38] Šimić G., Hof P. R. (2015). In search of the definitive Brodmann’s map of cortical areas in human. *Journal of Comparative Neurology*.

[39] Sun Y., Chan K., Krishnan S. M. (2002). ECG signal conditioning by morphological filtering. *Computers in Biology and Medicine*.

[40] Li Z., Leung J. Y., Tam E. W., Mak A. F. (2006). Wavelet analysis of skin blood oscillations in persons with spinal cord injury and able-bodied subjects. *Archives of Physical Medicine and Rehabilitation*.

[41] Farina D., do Nascimento O. F., Lucas M. F., Doncarli C. (2007). Optimization of wavelets for classification of movement-related cortical potentials generated by variation of force-related parameters. *Journal of Neuroscience Methods*.

[42] Chang C. C., Lin C. J. (2011). *LIBSVM: A Library for Support Vector Machines*.

[43] Lotze M., Flor H., Grodd W. (2001). Phantom movements and pain. An fMRI study in upper limb amputees. *Brain*.

[44] Wu J., Stoica B. A., Luo T. (2014). Isolated spinal cord contusion in rats induces chronic brain neuroinflammation, neurodegeneration, and cognitive impairment: involvement of cell cycle activation. *Cell Cycle*.

[45] Wu J., Zhao Z., Sabirzhanov B. (2014). Spinal cord injury causes brain inflammation associated with cognitive and affective changes: role of cell cycle pathways. *Journal of Neuroscience*.

[46] Mihara M., Miyai I., Hatakenaka M., Kubota K., Sakoda S. (2008). Role of the prefrontal cortex in human balance control. *Neuroimage*.

